# Maternal Systemic Lupus Erythematosus (SLE) High Risk for Preterm Delivery and Not for Long-Term Neurological Morbidity of the Offspring

**DOI:** 10.3390/jcm10132952

**Published:** 2021-06-30

**Authors:** Dora Davidov, Eyal Sheiner, Tamar Wainstock, Shayna Miodownik, Gali Pariente

**Affiliations:** 1 Soroka University Medical Center, Department of Obstetrics and Gynecology, Ben-Gurion University of the Negev, Beer-Sheva 84101, Israel; davidora88@gmail.com (D.D.); miodowni@post.bgu.ac.il (S.M.); galipa@bgu.ac.il (G.P.); 2 The Department of Public Health, Faculty of Health Sciences, Ben-Gurion University of the Negev, Beer-Sheva 84101, Israel; wainstoc@post.bgu.ac.il

**Keywords:** systemic lupus erythematosus, preterm delivery, neurologic morbidity, offspring

## Abstract

Objective: Pregnancies of women with systemic lupus erythematosus (SLE) are associated with preterm delivery. As preterm delivery is associated with long-term neurological morbidity, we opted to evaluate the long-term neurologic outcomes of offspring born to mothers with SLE regardless of gestational age. Methods: Perinatal outcomes and long-term neurological disease of children of women with and without SLE during pregnancy were evaluated. Children of women with and without SLE were followed until 18 years of age for neurological diseases. Generalized estimating equation (GEE) models were used to assess perinatal outcomes. To compare cumulative neurological morbidity incidence a Kaplan–Meier survival curve was used, and a Cox proportional hazards model was used to control for confounders. Result: A total of 243,682 deliveries were included, of which 100 (0.041%) were of women with SLE. Using a GEE model, maternal SLE was noted as an independent risk factor for preterm delivery. The cumulative incidence of long-term neurological disease was not found to be significantly higher when using the Kaplan Meier survival curves and maternal SLE was not found to be associated with long-term neurological disease of the offspring when a Cox model was used. Conclusion: Despite the association of SLE with preterm delivery, no difference in long-term neurological disease was found among children of women with or without SLE.

## 1. Introduction

Systemic lupus erythematosus (SLE) is a chronic multisystemic autoimmune inflammatory disease with a wide range of clinical manifestations and it affects many organ systems [1]. While SLE mainly affects young women of childbearing age, the prevalence varies with the sex, age and ethnicity [1,2,3]. Treatment of SLE focuses on preventing and decreasing the severity and duration of flares, with the use of NSAIDs and antimalarial drugs for mild to moderate SLE and high dose corticosteroids for severe disease [4,5]. 

Antiphospholipid antibodies (aPL) are the main predictors of pregnancy complications, including miscarriage, fetal death, prematurity and preeclampsia, with lupus anticoagulant (LAC) being strongly associated with miscarriage and late fetal loss [6,7,8]. Furthermore, infants of mothers with SLE who were exposed to anti-Ro/SSA or anti-La/SSB antibodies during pregnancy have shown an increased risk for neonatal lupus syndrome, with its most serious manifestation being fetal heart block [6,9,10]. 

Previous studies have examined immediate pregnancy outcomes of women with SLE. Recurrent pregnancy loss, preeclampsia, fetal growth restriction, preterm delivery, cesarean delivery and postpartum infections [9,11,12] are some of the obstetrical complications shown to be correlated with SLE. 

Studies have pointed out an association of long-term neurological diseases in children of mothers with SLE, such as neurodevelopment impairment, learning and speech disorder, attention deficit hyperactivity disorder (ADHD) and autism spectrum disorders [13,14,15]. However these studies were limited by smaller cohorts. 

Several factors have been suggested to affect neurodevelopment impairment among children born to mothers with SLE. This largely involves maternal autoantibodies that cross the placenta during fetal development which may result in learning disorders and dyslexia in offspring [16,17]. Additionally, prematurity was found to be linked to long-term neurological disease, such as cerebral palsy, particularly when chorioamnionitis was involved [18,19]. 

Due to the link between maternal SLE and preterm delivery, we opted to investigate the association between maternal SLE and unfavorable perinatal outcome, and long term neurological disease of children born to mothers with SLE, regardless of gestational age. 

## 2. Materials and Methods

This was a population-based retrospective cohort study that investigated perinatal results and long-term neurological diseases of children of mothers with and without SLE. The study compared various perinatal outcomes, such as perinatal mortality, caesarean delivery, recurrent pregnancy loss, hypertensive disorders, placental abruption and preterm delivery. A predefined set of ICD-9 codes (Table S1) was used as the basis for assessing the total neurological diseases of children of mothers with SLE up to the age of 18 years. These neurological diseases included psychiatric emotional disorders, movement disorders and total neurologic-related hospitalizations. Follow-up time was determined as time to an event (hospitalization with any neurological diagnosis). If any of the following occurred, follow-up ended: hospitalization resulting in death, first hospitalization with any neurological diagnosis or when the child reached 18 years of age. The study population included all children born to mothers with SLE during the years 1991–2014 at Soroka University Medical Center (SUMC), a tertiary hospital and the only medical center in the Negev, the southern region of the country, which covers 65% of the country’s area (about 1.22 million people) [20]. Multifetal pregnancies and cases of congenital anomalies and chromosomal abnormalities were excluded from the study. Cases of perinatal mortality were excluded from the long-term analysis. Pregnancies prior or after the study period (1991–2014) were not included in the study. Hence some of the women in our study were multipara but only part of their pregnancies was included in our study. The institutional review board, in accordance with Helsinki declaration, approved the study (IRB number 0357-19-SOR). 

Data were collected from two cross-linked and merged computerized databases, each based on mother and infant ID numbers: the perinatal database of the Obstetrics and Gynecology Department and the pediatric hospitalization database of SUMC (Demog-ICD9). The perinatal database contains maternal demographics, diseases and perinatal results documented immediately after delivery and anonymized before analysis. The pediatric hospitalization database contains demographic data and ICD-9 codes for all medical diagnoses recorded during any hospitalization at SUMC. 

Among 100 women with SLE, data regarding disease activity, manifestations, presence of anti-Ro/La antibodies, anti dsDNA antibodies and anti-phospholipid syndrome (APS) antibodies were gathered by examining each women’s file. The data were in 70% of the files (i.e., 30 women with SLE did not have information regarding disease activity or lack thereof during pregnancy). Severe SLE during pregnancy was defined as having active SLE during pregnancy (i.e., experiencing any SLE manifestation during pregnancy).

### Statistical Analysis

Statistical analysis was executed utilizing SPSS 23rd edition. To compare background characteristics between the two study groups, univariable analysis was performed which included *t*-tests or Mann-Whitney U tests for continuous variables and chi-square tests for categorical variables. Generalized estimating equation (GEE) models were used to compare perinatal outcomes, controlling for confounders and for maternal clusters. To compare the cumulative incidence of neurologic-related hospitalization in offspring of mothers with and without SLE a Kaplan-Meier survival curve was used. Finally, a Cox proportional hazards model was used to control for confounders. A *p*-value of ≤0.05 was considered statistically significant. All analyses were two-sided.

Based on initial analysis, after excluding the offspring who did not meet the inclusion criteria, there were 242,246 offspring born to mothers without SLE, and 96 offspring born to mothers with SLE. The rate of pediatric neurological-associated hospitalizations, based on initial analysis, was 4.2% (*n* = 4x). This sample size has a power of 80% to detect an odds ratio of 5.8% between the study groups.

## 3. Results

### 3.1. Maternal Characteristics and Perinatal Outcomes

This study included 243,682 deliveries that met the inclusion criteria, of which 100 were from women with SLE (0.041%). Information regarding disease activity during pregnancy was found in 70 women with SLE, of those, 20 women had flares during pregnancy. Disease activity manifested with arthritis (12 women), nephritis (9 women), dermatitis (8 women) and carditis (1 woman). Some women with flares had more than one clinical manifestation. While anti Ro/La antibodies were present in 3 women with flares during pregnancy, anti dsDNA antibodies were present in 10 women with SLE flares during pregnancy. Two women had prior APS antibodies. No information regarding prior organ damage was found in our population. Demographic and clinical characteristics of women with SLE are presented in Table 1.

Mothers with SLE were older and demonstrated higher rates of recurrent pregnancy loss. Perinatal outcomes of the study population are described in Table 2.

Higher rates of hypertensive disorders (13.0% vs. 5.0%, *p* < 0.001(caesarean delivery (40.0% vs. 13.5%, *p* < 0.001), preterm delivery (28.0% vs. 6.9%, *p* < 0.001) and perinatal mortality (4.0% vs. 0.5%, *p* < 0.001) were noted among women with SLE compared with women without SLE. 

A GEE model that controlled for maternal age and disorders of hypertension demonstrated that maternal SLE was an independent risk factor for preterm delivery (adjusted OR 4.9, 95% CI 3.20–7.80, *p* < 0.001). Using another GEE model controlling for gestational age, the association between maternal SLE and perinatal mortality lost its significance (adjusted OR 2.4, 95% CI 0.78–7.93, *p* = 0.123, Table 3).

### 3.2. Long Term Neurological Morbidity of Offspring to Mothers with SLE

After eliminating all cases of antepartum, intrapartum, and postpartum mortality, the population of the study included 242,342 children, among them 96 children of mothers with SLE. No significant difference was noted in long-term neurological disease between children to mothers with SLE and without SLE (4.2% vs. 3.1%, *p* = 0.552, Table 4).

Severe SLE during pregnancy was defined as having flares of SLE manifestations during pregnancy. No significant difference was noted in long-term neurological disease between children to mothers with severe SLE during pregnancy compared with children to mothers with SLE with no manifestation of severity during pregnancy (*p* = 0.79)

**Figure 1:**
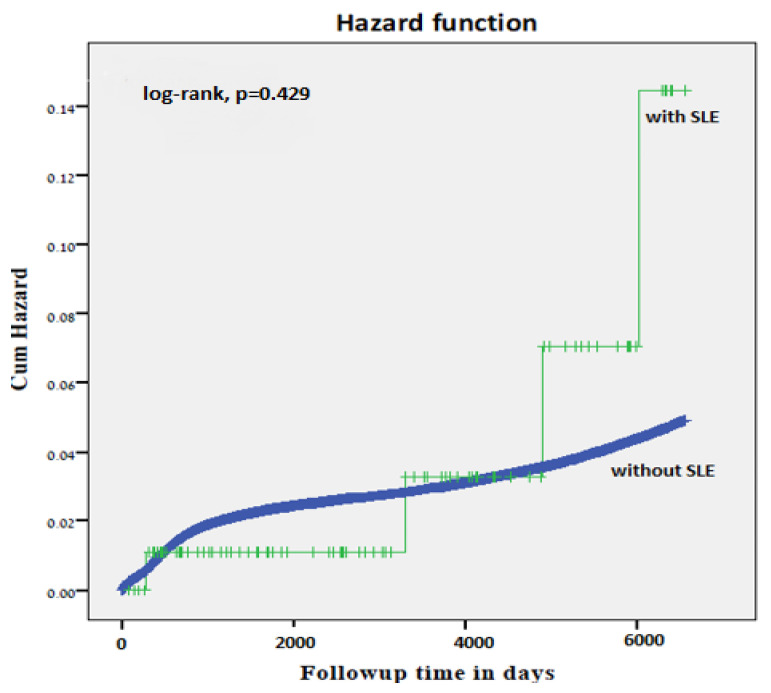
Kaplan-Meier survival curve demonstrating the cumulative incidence of neurologic-related hospitalizations in offspring of mothers with and without SLE (log-rank test, *p* = 0.429).

Similarly, no significantly higher cumulative incidence rate of long-term neurological morbidity in offspring of women with SLE was demonstrated by the Kaplan Meier survival curve (log-rank test *p* = 0.429, Figure 1).

A Cox proportional hazards model, controlling for gestational age and maternal age, demonstrated that being born to a mother with SLE was not found to be independently associated with long-term neurological disease of the offspring (adjusted HR 1.3, 95% CI 0.51–3.62, *p* = 0.539, Table 5).

## 4. Discussion

The major finding of our study is that while maternal SLE was associated with a significantly higher risk for adverse perinatal outcomes such as preterm delivery, no significant difference in long-term neurological morbidity was found between offspring born to mothers with or without SLE. 

### 4.1. Perinatal Outcomes of Mothers with SLE

The association between maternal SLE and adverse perinatal outcomes is well established. Studies of perinatal outcomes in women with SLE showed higher rates of recurrent pregnancy loss, preeclampsia, fetal growth restriction, cesarean delivery, postpartum infections and preterm delivery [12,21]. 

Our study supports these findings, as hypertensive disorders, cesarean delivery rates, and recurrent pregnancy loss were higher among mothers with SLE compered with mothers without SLE. Preterm delivery rates were also higher among mothers with SLE. As preterm delivery has been demonstrated to be associated with maternal age [22], and hypertensive disorders [23,24], after using a GEE model controlling for maternal age and hypertensive disorders, maternal SLE was independently associated with preterm delivery. 

### 4.2. Long Term Neurological Morbidity in Offspring Born to Mothers with SLE 

Contrary to our study, previous studies found an increased risk for long-term neurological morbidity in offspring of mothers with SLE, including learning disorders, ADHD and autism spectrum disorders [14,16,25,26]. Nalli, et al. [26] reported an increased risk of learning disorders in offspring to mothers positive for aPL antibodies. Nevertheless, a restricted sample size of only 10 children born to mothers with SLE, lack of a control group such as children born to mothers with systemic autoimmune diseases yet negative for aPL and self reported evaluations made it hard to interpret the study results. In their case-control study, Ross, et al. [16] studied 58 children born to mothers with SLE and 58 children born to healthy mothers. The authors demonstrated that sons of women with SLE, rather than daughters, were significantly more likely to have learning disabilities, and that the presence of anti-Ro and anti-La antibodies and disease activity were significantly related to a higher prevalence of learning disabilities in the offspring. As our study focused on neurological morbidity that is related to the health of the offspring, learning disabilities were not addressed in our study. 

Prior studies have demonstrated the influence of maternal autoantibodies on several aspects of fetal development during pregnancy, such as the presence of anti-Ro/anti-La antibodies and the increased risk of neonatal lupus and congenital heart block [13,17,27]. Other studies indicated that anti-La antibodies were associated with developmental delays in offspring [26,28].

Animal studies demonstrated that anti-dsDNA antibodies and anti-NMDR antibodies, which are both present in SLE patients, cross the placenta and influence fetal neurological development [29]. Surprisingly, our study did not demonstrate a significant association between maternal SLE and long-term neurological morbidity. Lack of association might be due to the fact that some neurological-related morbidities only manifest at ages older than 18 years. Another explanation may be related to the fact that most women with SLE in our population did not manifest symptoms of severe disease during pregnancy and only a minority had positive autoantibodies such as anti-Ro/La of dsDNA autoantibodies. To understand the association between maternal SLE and its long-term neurological effects on offspring, further studies should be done to assess the neurodevelopment spectrum of offspring from childhood to adolescence and differences in offspring outcome between women with and without severe SLE during pregnancy. 

### 4.3. Strengths and Limitations of the Study

Our study worked with a large population and with a long follow-up period, in an effort to evaluate the risk of neurologic-related hospitalizations in children born to mothers with SLE, thereby decreasing the chances of incorrect exposure and outcome data. By combining maternal, neonatal, and long-term childhood data, we were able to demonstrate the long-term outcomes of children, while controlling for several parameters during pregnancy and delivery. Healthcare in the country is universal, with all citizens provided equal and free medical care irrespective of their socio-economic standing. Inequity in medical access being reduced in such circumstances, it is unlikely that differences between social classes would be encountered. Nevertheless, our study has several limitations. First, our study lacked some clinical data regarding the severity of the disease, presence of specific antibodies and the treatment that was provided to mothers with SLE. However, as the two most common medications for treatment of SLE include antimalarial agents and corticosteroids, which have not been shown to effect long-term neurological development of the offspring, we would assume that the treatment provided to the mother would not influence the study’s results [30,31,32,33,34]. In addition to this no data regarding the reasons for the preterm deliveries or the reasons for the perinatal mortality cases were known to us, Secondly, because our study was based on hospitalizations alone, our data include only severe neurological morbidities and exclude minor morbidities that are mainly treated in regional clinics. Another limitation of our study is the rareness of both the exposure (maternal SLE) and outcome (long- term neurological morbidity of the offspring) in our population, which may result in lack of power to demonstrate a positive correlation between the two. Finally, since patients can choose other places of health care, patients may have chosen to be treated in health centers other than SUMC 

## 5. Conclusions

In conclusion, in our study population, maternal SLE does not appear to increase the risk for long-term neurologic hospitalizations in offspring. As most women with SLE during pregnancy in our population did not have severe disease during pregnancy and as the activity of the disease may influence the association between maternal SLE and long- term morbidity of the offspring, additional studies should investigate differences between mild to severe maternal SLE and their association with long- term neurological morbidity of the offspring. Further studies should also investigate the association between maternal SLE and other related neurodevelopmental concerns, in order to identify populations at need for long-term surveillance.

## Figures and Tables

**Table 1:** Demographic and clinical characteristics of mothers with and without SLE.

Characteristic	Mothers with SLE (*n* = 100)	Mothers without SLE (*n* = 243,582)	Odds Ratio (95% Confidence Interval)	*p*-Value
Maternal age, years (mean ± SD)	31.44 ± 4.62	28.16 ± 5.8	-	0.001
Gravidity (%) 1 2–4 ≥5				
	18.0	19.7		
	23.0	47.8	-	0.585
	59.0	32.5		
Parity (%) 1 2–4 ≥5				
	24.0	23.6		
	65.0	51.1	-	0.003
	11.0	25.3		
Recurrent pregnancy loss (%)	23.0	5.0	5.63 (3.53–8.98)	<0.001

**Table 2:** Perinatal outcomes of women with and without SLE.

Characteristic	Women with SLE (*n* = 100) (%)	Women without SLE (*n* = 243,582) (%)	Odds Ratio	95% CI	*p*-Value
Hypertensive disorders	13.0	5.0	2.8	1.57–5.06	<0.001
Placental abruption	1.0	0.6	1.8	0.25–12.92	0.552
Preterm delivery	28.0	6.9	5.2	3.41–8.18	<0.001
Caesarean delivery	40.0	13.5	4.2	2.85–6.35	<0.001
1 -Min Apgar score <7	8.0	5.3	1.5	0.75–3.18	0.258
5-Min Apgar score <7	3.0	2.3	1.3	0.42–4.22	0.496
Small for gestational age (SGA)	7.0	4.6	1.5	0.71–3.34	0.260
Perinatal mortality	4.0	0.5	7.5	2.77–20.57	<0.001

**Table 3:** GEE models for preterm delivery and perinatal mortality.

	Outcome		Adjusted OR	95% CI	*p*-Value
Model 1 *	Preterm Delivery	Maternal SLE (vs. no maternal SLE)	4.9	3.20–7.80	<0.001
Model 2 **	Perinatal mortality	Maternal SLE (vs. no maternal SLE)	2.4	0.78–7.39	0.123

* Model 1 controls for maternal age and hypertensive disorders. ** Model 2 controls for gestational age at birth.

**Table 4:** Selected long-term neurological morbidity in offspring of women with and without SLE.

Neurological Morbidity	Maternal SLE (*n* = 96)	No maternal SLE (*n* = 242,246)	*p*-Value
Movement disorder (%)	3.1	1.8	0.351
Psychiatric emotional disorder (%)	1.0	0.5	0.443
Total neurological hospitalizations (%)	4.2	3.1	0.552

**Table 5:** Long-term neurological morbidity of children to women with SLE assessed by Cox proportional hazards model.

	Adjusted HR	95% CI	*p* Value
Maternal SLE (vs. no maternal SLE)	1.3	0.51–3.62	0.539
Preterm delivery (<37 weeks)	1.5	1.41–1.65	<0.001
Mother Age at Birth	0.9	0.99–1.00	0.268

## Supplementary Materials

The following are available online at https://www.mdpi.com/article/10.3390/jcm10132952/s1, Table S1: neurological morbidities ICD-9 codes.
