# Supplementary material for: Maternal Systemic Lupus Erythematosus (SLE) High Risk for Preterm Delivery and Not for Long-Term Neurological Morbidity of the Offspring
Source: J Clin Med. 2021 Jun 30;10(13):2952. doi: 10.3390/jcm10132952 (PMC8269125; doi:10.3390/jcm10132952)
Supplement: Supplementary file 1 [file jcm-10-02952-s001.zip › jcm-1257047-supplementary.pdf]

Table S1: neurological morbidities ICD-9 codes	
**ICD-9 Code**	**Diagnosis**
3071	ANOREXIA NERVOSA
3075	OTHER AND UNSPECIFIED DISORDERS OF EATING
30750	EATING DISORDER, UNSPECIFIED
30751	BULIMIA NERVOSA
30753	RUMINATION DISORDER
30759	OTHER DISORDERS OF EATING
V691	INAPPROPRIATE DIET & EATING HABITS
3073	STEREOTYPIC MOVEMENT DISORDER
3331	ESSENTIAL AND OTHER SPECIFIED FORMS OF TREMOR
3332	MYOCLONUS
3335	OTHER CHOREAS
3336	GENETIC TORSION DYSTONIA
3336	IDIOPATHIC TORSION DYSTONIA
3343	OTHER CEREBELLAR ATAXIA
3450	GENERALIZED NONCONVULSIVE EPILEPSY
3452	PETIT MAL STATUS, EPILEPTIC
3453	GRAND MAL STATUS, EPILEPTIC
3455	PARTIAL EPILEPSY, WITHOUT IMPAIRMENT OF CONSCIOUSNESS
3456	INFANTILE SPASMS
3459	EPILEPSY, UNSPECIFIED
7810	ABNORMAL INVOLUNTARY MOVEMENTS
7812	ABNORMALITY OF GAIT
7813	LACK OF COORDINATION
33390	UNSP.EXTRAPYRAMIDAL DISEASE + ABNORMAL MOVEMENT DISORDER
33399	OTHER EXTRAPYRAMIDAL DISEASES AND ABNORMAL MOVEMENT DISORDERS
34500	GENERALIZED NONCONVULSIVE EPILEPSY WITHOUT INTRACTABLE EPILEPSY
34501	GENERALIZED NONCONVULSIVE EPILEPSY WITH INTRACTABLE EPILEPSY
34510	GENERALIZED CONVULSIVE EPILEPSY WITHOUT INTRACTABLE EPILEPSY
34511	GENERALIZED CONVULSIVE EPILEPSY WITH INTRACTABLE EPILEPSY
34540	PARTIAL EPILEPSY+IMPAIRMENT OF CONSCIOUSNESS WITHOUT INTRACTABLE EPILEPSY
34550	PARTIAL EPILEPSY WITHOUT IMPAIRMENT OF CONSCIOUSNESS WITHOUT INTR ACTABEL EPILEPSY
34560	INFANTILE SPASMS WITHOUT INTRACTABLE EPILEPSY
34590	EPILEPSY, NUSP. WITHOUT INTRACTABEL EPILEPSY
34590	EPILEPSY, UNSP. WITHOUT INTRACTABLE EPILEPSY
34591	EPILEPSY UNSP. WITH INTRACTABLE EPILEPSY
78031	FEBRILE CONVULSIONS

78031	FEBRILE CONVULSIONS (SIMPLE), UNSPECIFIED
78032	COMPLEX FEBRILE CONVULSIONS
78039	OTHER CONVULSIONS
78099	OTHER GENERAL SYMPTOMS
3341	HEREDITARY SPASTIC PARAPLEGIA
3421	SPASTIC HEMIPLEGIA
3429	HEMIPLEGIA, UNSPECIFIED
3430	CONGENITAL DIPLEGIA
3431	CONGENITAL HEMIPLEGIA
3432	CONGENITAL QUADRIPLEGIA
3439	INFANTILE CEREBRAL PALSY, UNSPECIFIED
3441	PARAPLEGIA
3442	DIPLEGIA OF UPPER LIMBS
3449	PARALYSIS, UNSPECIFIED
3481	ANOXIC BRAIN DAMAGE
3526	MULTIPLE CRANIAL NERVE PALSIES
7814	TRANSIENT PARALYSIS OF LIMB
34210	SPASTIC HEMIPLEGIA AFFECTING UNSP. SIDE
34290	HEMIPLEGIA, UNSP., AFFECTING UNSP. SIDE
34291	HEMIPLEGIA, UNSP., AFFECTING DOMINANT SIDE
34292	HEMIPLEGIA, UNSP., AFFECTING NONDOMINANT SIDE
34400	QUADRIPLEGIA, UNSPECIFIED
34430	MONOPLEGIA OF LOWER LIMB, AFFECTING UNSP. SIDE
34440	MONOPLEGIA OF UPPER LIMB, AFFECTING UNSP. SIDE
34489	OTHER SPECIFIED PARALYTIC SYNDROME
43811	APHASIA
43820	HEMIPLEGIA AFFECTING UNSP. SIDE
309	ADJUSTMENT REACTION
311	DEPRESSIVE DISORDER, NOT ELSEWHERE CLASSIFIED
316	PSYCHIC FACTORS ASSOCIATED WITH DISEASES CLASSIFIED ELSEWHERE
2930	ACUTE DELIRIUM
2930	DELIRIUM DUE TO CONDITIONS CLASSIFIED ELSEWHERE
2940	AMNESTIC DISORDER IN CONDITIONS CLASSIFIED ELSEWHERE
2949	UNSPECIFIED PERSISTENT MENTAL DISORDERS DUE TO COND.CLASS.ELSEWH.
2971	DELUSIONAL DISORDER
2979	UNSPECIFIED PARANOID STATE
2981	EXCITATIVE TYPE PSYCHOSIS
2983	ACUTE PARANOID REACTION
2989	UNSPECIFIED PSYCHOSIS
3003	OBSESSIVE-COMPULSIVE DISORDERS
3004	DYSTHYMIC DISORDER

3004	NEUROTIC DEPRESSION
3009	UNSPECIFIED NONPSYCHOTIC MENTAL DISORDER
3019	UNSPECIFIED PERSONALITY DISORDER
3026	DISORDERS OF PSYCHOSEXUAL IDENTITY
3051	TOBACCO USE DISORDER (TOBACCO DEPENDENCE)
3061	RESPIRATORY MALFUNCTION ARISING FROM MENTAL FACTORS
3062	CARDIOVASCULAR MALFUNCTION ARISING FROM MENTAL FACTORS
3068	OTHER SPECIFIED PSYCHOPHYSIOLOGICAL MALFUNCTION
3069	UNSPECIFIED PSYCHOPHYSIOLOGICAL MALFUNCTION
3070	ADULT ONSET FLUENCY DISORDER
3070	STAMMERING AND STUTTERING
3070	STUTTERING
3080	PREDOMINANT DISTURBANCE OF EMOTIONS
3089	UNSPECIFIED ACUTE REACTION TO STRESS
3090	ADJUSTMENT DISORDER WITH DEPRESSED MOOD
3094	ADJUSTMENT DISOR.WITH MIXED DISTURB.OF EMOTIONS AND CONDUCT
3099	UNSPECIFIED ADJUSTMENT REACTION
3129	UNSPECIFIED DISTURBANCE OF CONDUCT
3139	UNSPECIFIED EMOTIONAL DISTURBANCE OF CHILDHOOD OR ADOLESCENCE
7801	HALLUCINATIONS
7803	CONVULSIONS
7992	NERVOUSNESS
7993	DEBILITY, UNSPECIFIED
29384	ANXIETY DISORDER IN CONDITIONS CLASSIFIED ELSEWHERE
29530	PARANOID TYPE SCHIZOPHRENIA, UNSPECIFIED STATE
29570	SCHIZOAFFECTIVE DISORDER SCHIZOPHRENIA, UNSPECIFIED STATE
29580	OTHER SPECIFIED TYPES OF SCHIZOPHRENIA, UNSPECIFIED STATE
29590	UNSPECIFIED TYPE SCHIZOPHRENIA, UNSPECIFIED STATE
29600	BIPOLAR I DISORDER, SINGLE MANIC EPISODE, UNSPECIFIED DEGREE
29620	MAJOR DEPRESSIVE AFFECTIVE DISORDER,SINGLE EPISODE,UNSP.DEGREE
29680	BIPOLAR DISORDER, UNSPECIFIED
29690	UNSPECIFIED EPISODIC MOOD DISORDER
29699	OTHER SPECIFIED AFFECTIVE PSYCHOSES
30000	ANXIETY STATE, UNSPECIFIED
30001	PANIC DISORDER WITHOUT AGORAPHOBIA
30009	OTHER ANXIETY STATES
30010	HYSTERIA, UNSPECIFIED
30011	CONVERSION DISORDER
30029	OTHER ISOLATED OR SIMPLE PHOBIAS
30183	BORDERLINE PERSONALITY
30183	BORDERLINE PERSONALITY DISORDER

30302	AC.ALCOHOLIC INTOXIC.IN ALCOHOLISM,EPISODIC DRINKING BEHAVIOR
30400	OPIOID TYPE DEPENDENCE, UNSPECIFIED USE
30430	CANNABIS DEPENDENCE, UNSPECIFIED USE
30432	CANNABIS DEPENDENCE, EPISODIC USE
30500	ALCOHOL ABUSE, UNSPECIFIED DRINKING BEHAVIOR
30501	ALCOHOL ABUSE, CONTINUOUS DRINKING BEHAVIOR
30502	ALCOHOL ABUSE, EPISODIC DRINKING BEHAVIOR
30591	OTHER, MIXED, OR UNSPECIFIED DRUG ABUSE, CONTINUOUS USE
30720	TIC DISORDER, UNSPECIFIED
30722	CHRONIC MOTOR OR VOCAL TIC DISORDER
30723	TOURETTE'S DISORDER
30752	PICA
30924	ADJUSTMENT DISORDER WITH ANXIETY
30981	POSTTRAUMATIC STRESS DISORDER
31210	UNDERSOCIALIZED CONDUCT DISORDER,UNAGGRESSIVE TYPE,UNSPECIFIED
31239	OTHER DISORDERS OF IMPULSE CONTROL
31389	OTHER EMOTIONAL DISTURBANCES OF CHILDHOOD OR ADOLESCENCE
79921	NERVOUSNESS
79922	IRRITABILITY
79925	DEMORALIZATION AND APATHY
79929	OTHER SIGNS AND SYMPTOMS INVOLVING EMOTIONAL STATE
V6284	SUICIDAL IDEATION
3142	HYPERKINETIC CONDUCT DISORDER OF CHILDHOOD
3149	UNSPECIFIED HYPERKINETIC SYNDROME OF CHILDHOOD
31400	ATTENTION DEFICIT DISORDER WITHOUT HYPERACTIVITY
31401	ATTENTION DEFICIT DISORDER WITH HYPERACTIVITY
V400	MENTAL AND BEHAVIORAL PROBLEMS WITH LEARNING
V409	UNSPECIFIED MENTAL OR BEHAVIORAL PROBLEM
317	MILD INTELLECUTAL DISABILITIES
317	MILD MENTAL RETARDATION
319	UNSPECIFIED INTELLECTUAL DISABILITIES
319	UNSPECIFIED MENTAL RETARDATION
3152	OTHER SPECIFIC DEVELOPMENTAL LEARNING DIFFICULTIES
3154	DEVELOPMENTAL COORDINATION DISORDER
3158	OTHER SPECIFIED DELAYS IN DEVELOPMENT
3159	UNSPECIFIED DELAY IN DEVELOPMENT
7834	LACK OF EXPECTED NORMAL PHYSIOLOGICAL DEVELOPMENT
7834	LACK OF EXPECTED NORMAL PHYSIOLOGICAL DEVELOPMENT IN CHILDHOOD
31531	EXPRESSIVE LANGUAGE DISORDER
31534	SPEECH AND LANGUAGE DEVELOPMENTAL DELAY DUE TO HEARING LOSS
31539	OTHER DEVELOPMENTAL SPEECH DISORDER

33183	MILD COGNITIVE IMPAIRMENT, SO STATED
78340	LACK OF NORMAL PHYSIOLOGICAL DEVELOPMENT, UNSPECIFIED
330	CEREBRAL DEGENERATIONS USUALLY MANIFEST IN CHILDHOOD
335	ANTERIOR HORN CELL DISEASE
340	MULTIPLE SCLEROSIS
3300	LEUKODYSTROPHY
3308	OTHER SPECIFIED CEREBRAL DEGENERATIONS IN CHILDHOOD
3313	COMMUNICATING HYDROCEPHALUS
3314	OBSTRUCTIVE HYDROCEPHALUS
3319	CEREBRAL DEGENERATION, UNSPECIFIED
3348	OTHER SPINOCEREBELLAR DISEASES
3350	WERDNIG-HOFFMANN DISEASE
3360	SYRINGOMYELIA AND SYRINGOBULBIA
3410	NEUROMYELITIS OPTICA
3411	SCHILDER'S DISEASE
3419	DEMYELINATING DISEASE OF CENTRAL NERVOUS SYSTEM, UNSPECIFIED
3480	CEREBRAL CYSTS
3590	CONGENITAL HEREDITARY MUSCULAR DYSTROPHY
3591	HEREDITARY PROGRESSIVE MUSCULAR DYSTROPHY
33189	OTHER CEREBRAL DEGENERATION
33510	SPINAL MUSCULAR ATROPHY, UNSPECIFIED
33522	PROGRESSIVE BULBAR PALSY
33523	PSEUDOBULBAR PALSY
34120	ACUTE (TRANSVERSE) MYELITIS NOS
348891	CEREBRAL CALCIFICATION
3313 2	POST HEMORRHAGIC HYDROCEPHALUS
352	DISORDERS OF OTHER CRANIAL NERVES
3379	UNSPECIFIED DISORDER OF AUTONOMIC NERVOUS SYSTEM
3510	BELL'S PALSY
3518	OTHER FACIAL NERVE DISORDERS
3519	FACIAL NERVE DISORDER, UNSPECIFIED
3539	UNSPECIFIED NERVE ROOT AND PLEXUS DISORDER
3542	LESION OF ULNAR NERVE
3548	OTHER MONONEURITIS OF UPPER LIMB
3549	MONONEURITIS OF UPPER LIMB, UNSPECIFIED
3553	LESION OF LATERAL POPLITEAL NERVE
3556	LESION OF PLANTAR NERVE
3558	MONONEURITIS OF LOWER LIMB, UNSPECIFIED
3559	MONONEURITIS OF UNSPECIFIED SITE
3562	HEREDITARY SENSORY NEUROPATHY
3564	IDIOPATHIC PROGRESSIVE POLYNEUROPATHY

3568	OTHER SPECIFIED IDIOPATHIC PERIPHERAL NEUROPATHY
3569	UNSPECIFIED IDIOPATHIC PERIPHERAL NEUROPATHY
3570	ACUTE INFECTIVE POLYNEURITIS
3571	POLYNEUROPATHY IN COLLAGEN VASCULAR DISEASE
3572	POLYNEUROPATHY IN DIABETES
3577	POLYNEUROPATHY DUE TO OTHER TOXIC AGENTS
3588	OTHER SPECIFIED MYONEURAL DISORDERS
3589	MYONEURAL DISORDERS, UNSPECIFIED
3592	MYOTONIC DISORDERS
3599	MYOPATHY, UNSPECIFIED
33709	OTHER IDIOPATHIC PERIPHERAL AUTONOMIC NEUROPATHY
33720	REFLEX SYMPATHETIC DYSTROPHY, UNSPECIFIED
33721	REFLEX SYMPATHETIC DYSTROPHY OF UPPER LIMB
33722	REFLEX SYMPATHETIC DYSTROPHY OF LOWER LIMB
35781	CHRONIC INFLAMMATORY DEMYELINATING POLYNEURITIS
35800	MYASTHENIA GRAVIS WITHOUT (ACUTE) EXACERBATION
3383	NEOPLASM RELATED PAIN (ACUTE) (CHRONIC)
3384	CHRONIC PAIN SYNDROME
3482	BENIGN INTRACRANIAL HYPERTENSION
3483	ENCEPHALOPATHY, UNSPECIFIED
3483	ENCEPHALOPATHY,NOT ELSEWHERE CLASSIFIED
3490	REACTION TO SPINAL OR LUMBAR PUNCTURE
3492	DISORDERS OF MENINGES, NOT ELSEWHERE CLASSIFIED
3499	UNSPECIFIED DISORDERS OF NERVOUS SYSTEM
3561	PERONEAL MUSCULAR ATROPHY
7802	SYNCOPE AND COLLAPSE
7843	APHASIA
30789	OTHER PSYCHALGIA
33381	BLEPHAROSPASM
33819	OTHER ACUTE PAIN
33829	OTHER CHRONIC PAIN
33903	EPISODIC PAROXYSMAL HEMICRANIA
34830	ENCEPHALOPATHY, UNSPECIFIED
34831	METABOLIC ENCEPHALOPATHY
34881	TEMPORAL SCLEROSIS
34889	OTHER CONDITIONS OF BRAIN
34981	CEREBROSPINAL FLUID RHINORRHEA
34989	OTHER SPECIFIED DISORDERS OF NERVOUS SYSTEM
78093	MEMORY LOSS
99701	CENTRAL NERVOUS SYSTEM COMPLICATION
99709	OTHER NERVOUS SYSTEM COMPLICATIONS
